# Social, economic, and political events affect gender equity in China, Nepal, and Nicaragua: a matched, interrupted time-series study

**DOI:** 10.1080/16549716.2020.1712147

**Published:** 2020-01-15

**Authors:** Tuan T. Nguyen, Ashley Darnell, Amy Weissman, Edward A. Frongillo, Roger Mathisen, Karin Lapping, Timothy D. Mastro, Mellissa Withers

**Affiliations:** a Alive & Thrive Southeast Asia, FHI 360, Hanoi, Vietnam; b Asia Pacific Regional Office (APRO), FHI 360, Bangkok, Thailand; c Arnold School of Public Health, University of South Carolina, Columbia, SC, USA; d Alive & Thrive, FHI 360, Washington, DC, USA; e Chief Science Office, FHI 360, Durham, NC, USA; f Keck School of Medicine, University of Southern California, Los Angeles, CA, USA

**Keywords:** China, gender equity, Gender Gap Index (GGI), health disparities/inequities, interrupted time-series analysis, Nepal, Nicaragua

## Abstract

**Background**: Progress in gender equity can improve health at the individual and country levels.

**Objectives**: This study’s objective was to analyze recent trends in gender equity and identify historical and contextual factors that contributed to changes in gender equity in three countries: China, Nepal, and Nicaragua.

**Methods**: To assess gender equity trends, we used the Gender Gap Index (GGI) from the World Economic Forum’s Global Gender Gap Report (2006–2017). The GGI incorporated data on economic participation, educational attainment, health, and political empowerment for almost 150 countries. We selected China, Nepal, and Nicaragua because of their major changes in GGI and diversity in geographical location and economic status. We reviewed major social, economic, and political events during 2006–2017, and identified key events in each country. We compared countries’ GGI with matched controls average using interrupted time-series analysis.

**Results**: Nepal and Nicaragua both had dramatic increases in GGI (improvement in equity), Nepal (β = 0.029; 95% CI: 0.003, 0.056) and Nicaragua (β = 0.035; 95% CI: 0.005, 0.065). This was strongly influenced by political empowerment, which likely impacted access to education and employment opportunities. Despite major economic growth and new policies to address gender inequities (e.g. the One-Child Policy), China saw a significant decline in GGI between 2010 and 2017 (β = −0.014; 95% CI: −0.024, −0.004), largely resulting from decreased gender equity in educational attainment, economic participation, and health/survival sub-indices.

**Conclusions**: Key social, economic, and political events helped explain trends in countries’ gender equity. Our study suggested that supportive social and political environments would play important roles in empowering women, which would advance human rights and promote health and well-being of individuals, households, communities, and countries.

## Background

Over the last two decades, dramatic improvements have been achieved globally in education, health, and economic growth. According to the World Bank, between 1990 and 2013, one billion people were lifted out of extreme poverty [1,2]. Yet, these improvements have not typically benefitted women as much as men, especially those in the lowest socioeconomic strata [3]. Due to gender inequities, women are at heightened vulnerability for a range of negative outcomes. Every day, around the world approximately 830 women die from preventable causes relating to pregnancy and childbirth [4]. In 2013, women represented two-thirds of the 757 million adults who were unable to read or write [5]. Although women contribute largely to socioeconomic development (e.g. 40% of the global labor force, 43% agricultural labor force, and ~50% of world’s university students) [5], they make up 60% of the world’s poorest [6]. About 19% of women’s time is spent on non-salaried activities such as taking care of the household as opposed to only 8% of men’s time [5]. The progress toward political empowerment has also been slow for women. For example, while the proportion of women in parliament positions rose 6% between 2006 and 2016, women still only occupy 23% of parliamentary seats globally [5].

Improved gender equity is critical to improve quality of life and is a focus of the United Nations Sustainable Development Goals (SDGs) that have been endorsed by almost all member states [5]. The increase in the female participation and reduction in the gender gaps in formal labor force participation result in faster economic growth that benefits individuals, households, and nations [5]. Eliminating barriers that disadvantage women, such as social norms that emphasize female household responsibilities like cooking, cleaning, and child care, could increase labor productivity by as much as 25% in some countries [1]. Additionally, greater control over household resources by women change spending patterns to beneﬁt children, and thus could enhance countries’ socio-economic development [1].

Although it is expected that a change in legislation and policies or major political, economic, and social events can have a direct influence on certain aspects of gender equity, it is still not known whether these would affect a complex gender index. Furthermore, the strength of the association may vary across countries due to differences in national and regional dynamics sociocultural norms. To address this knowledge gap, we conducted this study to (1) analyze recent trends in gender equity and (2) identify potential historical and contextual factors that may have contributed to changes in gender equity in three diverse country contexts – China, Nepal, and Nicaragua.

## Methods

Our study was based on Bernal’s six steps for conducting interrupted time-series analysis (ITSA) [7]. ITSA is a study design for evaluating the effectiveness of population-level interventions such as the introduction of new vaccines, cycle helmet legislation, and precautions against nosocomial infections, as well as the evaluation of health impacts of unplanned events such as the global financial crisis [7–9]. Although randomized control trials have long been considered the gold standard in evaluating the effectiveness of an intervention, they are generally not well suited for policies, programs, and events affecting the whole population [7]. We integrated information about the selection of countries, outcome and exposure variables, and conducted the following six-step statistical analysis.

### Evaluate the appropriateness of interrupted time series design (step 1)

#### Outcome variable

We used the Global Gender Gap Index (GGI) [10–21] to assess gender equity over time. The GGI score ranges from 0 to 100: higher GGI scores signify smaller gaps between females and males. The Global Gender Gap Report, produced annually since 2006 by the World Economic Forum, examines women’s achievements as compared to their male counterparts in almost 150 countries. It includes a Global GGI, created using four thematic dimensions: (1) economic participation and opportunity, (2) educational attainment, (3) health and survival, and (4) political empowerment [10]. Each component is assessed based on relevant indicators drawn from international databases such as the International Labor Organization, United Nations Development Programme, United Nations Educational, Scientific and Cultural Organization, World Economic Forum, CIA World Factbook, and Inter-Parliamentary Union [10].

Using PowerQuery function in Microsoft Excel 2016, we extracted GGI data from 2006 to 2017 from Table 3 of the World Economic Forum’s Global Gender Gap Reports. We combined the data and arranged it by country, year, and indicators to create a dataset using PowerQuery, and imported the data to Stata Version 15 for further analyses.

#### The studied countries and events

We explored GGI trends between 2006 and 2017 for almost 150 countries and visually identified 53 countries with shifts in GGI (Appendix 1). From those 53 countries, we purposely selected China, Nepal, and Nicaragua because: (1) they had substantial shifts in GGIs [10–21]; (2) they were diverse in geographical location, political, economic, population, and health status [1,22,23] (Appendix 2), and (3) at least one co-author had experience working in the country, allowing an insider’s view on the events and social climate in the selected countries.

To examine potential factors underlying the changes, we reviewed major social, economic, and political events from 2006 to 2017 from the World Economic Forum’s Global Gender Gap Reports from 2006 to 2017 [10–21] and the websites of BBC [24–26] with some additional information from the websites of CIA Factbook [23] and the World Bank [27] (Appendix 3). Then, we identified the key events that happened prior to the shifts in CGI that may explain the changes. For example, examining GGI trends, we found major shifts in GGIs in China in 2010; Nepal in 2009, 2011, and 2014; and Nicaragua in 2009 and 2012. Appendix 3 suggested economic-related events preceded GGI shifts in 2010 in China; political-related events preceded GGI shifts in 2009, 2011, and 2014 in Nepal; and economic- and political-related events preceded GGI shifts in 2009 and 2012 in Nicaragua.

### Proposing the impact model (step 2)

Because of the complexity of the GGI indicator as well as the events identified, we did not have a predetermined impact model (e.g. lag time, the change in slope, level, or direction). We refined the analysis after our descriptive and regression analyses (steps 3 and 4).

### Descriptive analysis (step 3)

We conducted a descriptive analysis which explored the trends of the GGI and their sub-indices.

### Regression analysis (step 4)

We used single interrupted time-series analysis using ITSA procedure. The ITSA procedure examines and compares the slopes and levels of the regressions before and after the event, which is considered a strength over a traditional ecological approach [28]. The lag time of one was identified using ACTEST procedure [29] with P-value < 0.05. Events were in 2010 in China; 2009, 2011, and 2014 in Nepal; and 2009 and 2012 in Nicaragua.

### Addressing methodological issues (step 5)

The limitation of the single-group, interrupted-time series analysis, however, was that unmeasured or unknown, time-varying confounders could affect the findings [28]. To address this limitation, we used matched, interrupted time-series analysis [30]. For each country, we identified matched controls – countries that had similar slope and level of GGI prior to the event compared with the studied country – using matched ITSA procedure. We then compared the slopes and levels of GGI between the studied country and their matched controls average, using the ITSA procedure. Having matched controls with (1) similar GGI compared with a studied country prior the event, and (2) different GGI after an event unique to the studied countries, helped to strengthen the causation inference between the event and the change in GGI, especially in comparison with the traditional ecological approach [30].

Using a P-value of 0.20 (i.e. recommended by Linden A to allow a certain level of variations to find controls) [30], the five matched control countries for China prior to 2010 were Honduras, Nicaragua, Paraguay, Peru, and Uruguay. The seven for Nepal prior to 2014 were Bahrain, Benin, Burkina Faso, Cameroon, Czech Republic, Ethiopia, and Turkey. The 16 for Nicaragua prior to 2009 were Curaçao, Malawi, Chile, The Gambia, Italy, Liberia, Sierra Leone, Peru, Greece, Brazil, Zimbabwe, China, Malta, Myanmar, Kenya, and Uruguay. Fewer events than ITSA were used in the matched, time-series analysis because the matched ITSA procedure could not identify matched countries for Nepal prior to 2009 and 2011 or for Nicaragua prior to 2012.

### Model checking and sensitivity analysis (step 6)

We conducted model checking and sensitivity analysis which we integrated in steps 4 and 5 to find the best fit models (e.g. selection of lag time in step 4 and selection of different years of event in step 5). We also examined the trends of the GGI sub-indices (e.g. economic participation, educational attainment, health, and political empowerment) to explain changes.

## Results

### GGI levels and slopes

**Figure 1.**
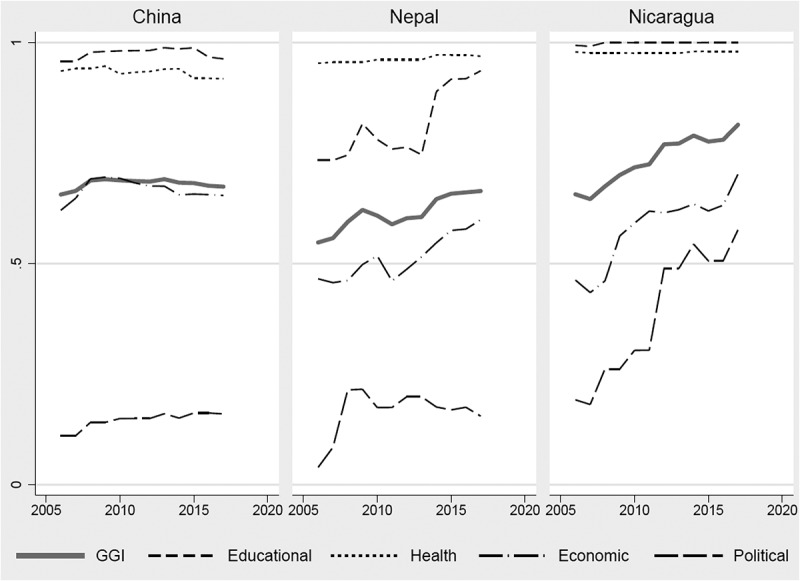
The trends of the gender gap index and sub-indices

In 2017, Nicaragua, China, and Nepal ranked 6, 100, and 111 out of 144 countries on the GGI, respectively [21]. China’s GGI score increased from ~0.65 in 2006 to ~0.69 in 2009, but then gradually decreased to about ~0.67 in 2017 (Figure 1). Between 2006 and 2017, Nepal’s GGI scores fluctuated between ~0.55 and 0.66, while Nicaragua’s GGI scores increased from 0.66 to 0.81 (Figure 1). Attributions to the change in the GGI in China were decreased equity in economic participation and opportunity, in Nepal they were improved educational and economic equity, and in Nicaragua, they were increased economic and political equity (Figure 1).

### Overview of events during the years with GGI shifts

There were two potential events in 2010 in China that might be associated with the changes in GGI: the global economic crisis and the residual impact of the One Child Policy. In Nepal, key events included the interim constitution was dissolved, negating the mandated quota system for political representation of women (2009), the withdrawal of the United Nations’ peacekeeping mission, which indicated more stability in the country (2011), and a new prime minister was elected and economic expansion was guaranteed with the hydropower dam deal with India (2014). In Nicaragua, the key events of 2012 were related to new legislation, including a law requiring 50% of political party candidates be female, and a law that better addressed gender-based violence (Appendix 3).

### Associations between events and GGI shifts

**Figure 2.**
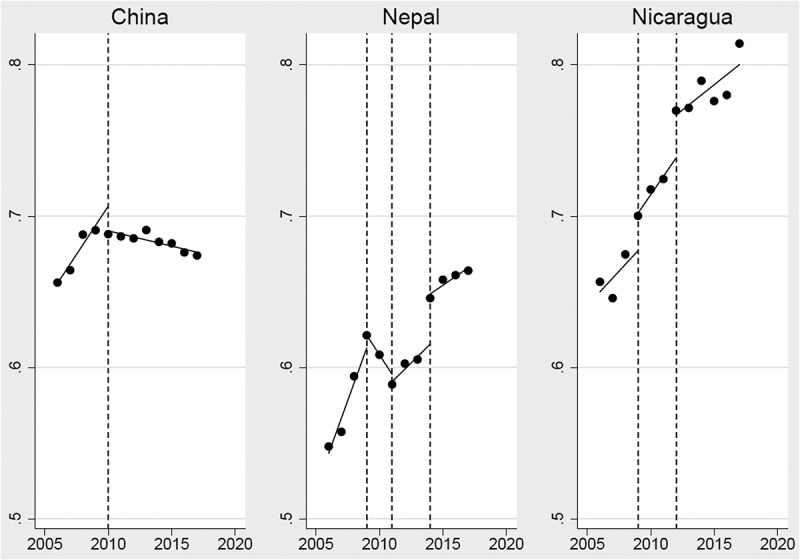
Association between key events and the gender gap index trend from 2006 to 2017: single-group, interrupted time-series analysis. Gender gap index (black dot) and predicted trend (solid line) by key event (vertical dash line)

In China, the GGI score decreased in level (β = −0.016; 95% CI: −0.026, −0.007) and slope (β = −0.015; 95% CI: −0.018, −0.011) after the event in 2009 (Table 1, Figure 2). In Nepal, the GGI score decreased in the slope (β = −0.016; 95% CI: −0.026, −0.007) after the event in 2009, but then increased in the slope after the 2011 events (β = 0.021; 95% CI: 0.015, 0.027) and leveled after the 2014 event (β = 0.033; 95% CI: 0.021, 0.045) (Table 1, Figure 2). Nicaragua had an increase in slope after the 2012 event (β = 0.028; 95% CI: 0.013, 0.044) (Table 1, Figure 2).

**Table 1.** Association between key events and the gender gap index trend from 2006 to 2017: single-group interrupted time-series analysis.^a^

	China	Nepal	Nicaragua
Number of observations	12	12	12
Intercept (β_0_)	0.656***	0.543***	0.650***
	(0.651, 0.660)	(0.525, 0.562)	(0.631, 0.669)
Slope before event 1 (β_1_)	0.013***	0.023*	0.009
	(0.010, 0.016)	(0.008, 0.039)	(−0.007, 0.025)
Change in level immediately after event 1 (β_2.1_)	−0.016**	0.008	0.025
	(−0.026, −0.007)	(−0.024, 0.041)	(−0.012, 0.061)
Difference in slopes between pre- and post- event 1 (β_3.1_)	−0.015***	−0.036**	0.003
	(−0.018, −0.011)	(−0.051, −0.021)	(−0.013, 0.019)
Change in level immediately after event 2 (β_2.2_)		−0.005	0.028**
		(−0.012, 0.003)	(0.013, 0.044)
Difference in slopes between pre- and post- event 2 (β_3.2_)		0.021***	−0.005
		(0.015, 0.027)	(−0.013, 0.002)
Change in level immediately after event 3 (β_2.3_)		0.033**	
		(0.021, 0.045)	
Difference in slopes between pre- and post- event 3 (β_3.3_)		−0.002	
		(−0.011, 0.006)	

Values are coefficient of regression (β) and 95% CIs. Significantly different from the null value (β = 0; two-sided t-tests): *P < 0.05, **P < 0.01, ***P < 0.001. Findings were generated using single-group, interrupted time-series analysis.

^a^Events were in 2010 in China; in 2009 and 2012 in Nicaragua; and in 2009, 2011, and 2014 in Nepal.

**Figure 3.**
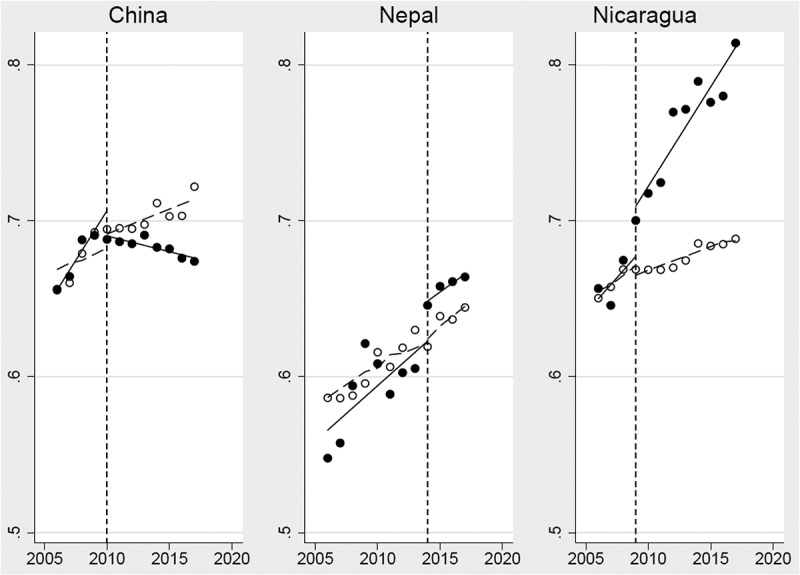
Association between key events and the gender gap index trend from 2006 to 2017: matched, interrupted time-series analysis. Gender gap index (black dot) and predicted trend (solid line) and of the controls’ average (hollow dot) and predicted trend (long-dash line), by key event (vertical dash line)

We found GGI levels and slopes of the studied countries were similar to their matched controls before the events in China in 2010, Nepal in 2014, and Nicaragua in 2009 (Table 2, Figure 3). After the events, China saw significant declines in the GGI slope (β = −0.014; 95% CI: −0.024, −0.004), while the other two countries had dramatic increases in GGI: Nepal (β = 0.029; 95% CI: 0.003, 0.056) and Nicaragua (β = 0.035; 95% CI: 0.005, 0.065) (Table 2, Figure 3). Compared with matched controls, linear post-event trends of the GGI in China had an annual decrease of 0.005 (95% CI: −0.01, −0.001) while in Nicaragua they had an annual increase of 0.010 (95% CI: 0.006, 0.013) (Table 2, Figure 3).

**Table 2.** Association between key events and the gender gap index trend from 2006 to 2017: matched, interrupted time-series analysis.^a,b^

	China	Nepal	Nicaragua
Number of observations	92	90	289
Intercept of the control group (β_0_)	0.669***	0.576***	0.655***
	(0.651, 0.686)	(0.568, 0.584)	(0.646, 0.663)
Slope before event of the control group (β_1_)	0.004	0.007***	0.005**
	(−0.005, 0.013)	(0.005, 0.009)	(0.001, 0.009)
Difference in the level between the studied country and control group before event (β_4_)	−0.013	−0.010	−0.005
	(−0.031, 0.005)	(−0.039, 0.019)	(−0.019, 0.010)
Difference in slopes between the studied country and control group before event (β_5_)	0.009	−0.000	0.004
	(−0.000, 0.018)	(−0.006, 0.005)	(−0.006, 0.014)
Change in level immediately after event of control group (β_2_)	0.009	−0.004	−0.003
	(−0.016, 0.034)	(−0.019, 0.012)	(−0.018, 0.012)
Difference in the slopes between pre- and post- event of the control group(β_3_)	−0.001	−0.001	−0.002
	(−0.011, 0.009)	(−0.008, 0.007)	(−0.006, 0.002)
Difference in the level between the studied country and control group immediately following event (β_6_)	−0.025	0.029*	0.035*
	(−0.051, 0.001)	(0.003, 0.056)	(0.005, 0.065)
Difference between the studied country in the slope after event compared with before event (β_7_)	−0.014**	−0.001	0.006
	(−0.024, −0.004)	(−0.010, 0.009)	(−0.004, 0.016)
Linear post-event trends			
Studied country	−0.002***	0.006***	0.013***
	(−0.003, −0.001)	(0.004, 0.008)	(0.01, 0.016)
Controls	0.003	0.006	0.003*
	(−0.002, 0.008)	(−0.001, 0.014)	(0.001, 0.005)
Difference	−0.005*	−0.001	0.010***
	(−0.01, −0.001)	(−0.008, 0.007)	(0.006, 0.013)

Values are coefficient of regression (β) and 95% CIs. Significantly different from the null value (β = 0; two-sided t-tests): *P < 0.05, **P < 0.01, ***P < 0.001. Findings were generated using matched, interrupted time-series analysis. Matched controls were selected by matching the level and trend of gender gap index of the studied countries before the event with those of other 146 countries with the estimation of gender gap index.

^a^Events were in 2010 in China, in 2014 Nepal, and in 2009 in Nicaragua.

^b^Matched control countries: for ***China (5 countries)***: Honduras, Nicaragua, Paraguay, Peru, and Uruguay; for ***Nepal (7 countries)***: Bahrain, Benin, Burkina Faso, Cameroon, Czech Republic, Ethiopia, Turkey; and for ***Nicaragua (16 countries)***: Curaçao, Malawi, Chile, The Gambia, Italy, Liberia, Sierra Leone, Peru, Greece, Brazil, Zimbabwe, China, Malta, Myanmar, Kenya, Uruguay.

## Discussion

Using a data-driven, analytical method through a matched, interrupted time-series analysis, we found that social, economic, and political shifts could help explain changes in gender equity. The associations differed by country. For example, we found a decreased GGI in China, driven by economic policies, and increased GGIs in Nepal and Nicaragua, which were associated with policies promoting education and employment opportunities for women.

China achieved a 17.7% rise in exports between 2010 and 2018 and soon became one of the world’s largest economies [24]. China’s progress towards gender parity increased between 2006 and 2010 but then decreased after [10–21], which can be attributed to four factors. First, there was a push for job creation through infrastructure development, such as new construction and building roads, which is historically a male-dominated [31,32]. Second, there was a substantial wage gap between women and men in China in both urban and rural areas [32,33]. Third, gender equity gaps in labor also widened because of gender discrimination in job opportunities [34,35]. Recognizing the challenges the country is facing, with the objective of keeping women in the workforce, nine government ministries and groups in China released a notice to regulate the recruitment and to promote employment among women, which detailed the violations and enforcement mechanisms [36]. Fourth, China has the world’s highest male-to-female ratio at birth, which lowered its GGI [13,14]. The relaxation of the One-Child Policy and legislation prohibiting sex-selective practices are major steps but it would take time to regain the gender balance, especially as China is a country with male preference cultural norms [37]. China’s gender imbalance contributed to slow population and labor force growth, increased numbers of single men, trafficking of women from other countries, and rising crime rates [38].

Nepal’s recent political history has been tumultuous, including a decade-long civil war (1996–2006) and transition toward a republic [25]. In 2015, a massive earthquake hit Nepal, killing more than 8,000 people, causing mass devastation, and leaving millions homeless and destitute [25]. During 2006–2017, an overall improved trend in the GGI might have resulted from political stability and policies or commitments in favor of women. They may be attributed to better access to girls’ education, which was encouraging given that Nepal’s cultural norms have historically limited girls’ access to education and hindered women’s empowerment [39]. The new constitution recognized the rights of women, including rights to lineage, safe maternity and reproduction, freedom from all forms of exploitation and discrimination, and equal rights in family matters and property [40,41]. The government also required more gender parity in terms of composition of the cabinet, setting a quota to increase women’s presence in the cabinet and legislative structures [42]. It also promised to work to end child marriage by 2030 [40].

Nicaragua was among the best performers in terms of GGI in the world and had a dramatic increase in its overall GGI ranking from 66th in 2006 to 6th in 2017 [10,21]. The latest rise is primarily due to improvements in gender parity on economic and political indices [16,21]. Legislation (2012) required that women comprise 50% of political party candidates [43], which helped Nicaragua to improve gender parity in the cabinet and parliament [18] resulting in more female legislators, ministers, lawmakers, senior officials, and managers [21]. In 2012, a comprehensive law addressing gender-based violence (i.e. femicide, economic and psychological violence against women) passed but was overturned in just two years [43]. The strong commitment to women rights and female political empowerment helped to increase occupational opportunities for females and maintain high level of gender equity in health and education, which can explain the two spikes in GGI in 2009 and 2012. However, this progress has been eroding ever since [43]. Despite narrowing the gap between males and females in GGI and its sub-indices, Nicaragua is not performing well on economic, health, education, and social issues [1,22,23,44,45]. Also, it is one of the only six countries in the world to restrict abortion without exception [26,43].

Our overall findings suggest that supportive social and political environments are central to ensuring better health and increased opportunities for women [2]. SDG 5 on gender equality calls for equal opportunities for female leadership and full participation ‘at all levels of decision-making in political, economic and public life’ [46]. It is recognized that the current low levels of political participation among women [47] undermine the ability to achieve this goal by 2030 [46]. Gender-related norms contribute to low female political participation due to expectations that women perform domestic, unpaid work such as taking care of their families and households [47–51]. Increasing women’s voice in other areas, such as trade unions, corporations, community groups, and professional associations can also help foster greater leadership roles for women [47]. It is important to consider the wide-reaching gender-related consequences of political changes, economic growth, and legislative opportunities to maximize the likelihood that these will improve, not impede, gender equity.

Our results also suggest that increasing quality employment opportunities for women is crucial to achieving gender equity. Improvements in women’s economic participation were found to be a common factor associated with GGI changes across the countries in our study. Gender inequity in employment relating to job opportunities, salaries, high-skilled positions, access to further education, unemployment rates, and unpaid work worldwide demonstrates the persistence of sociocultural norms which perpetuate gender inequities in this arena [50,51]. A focus on the creation of jobs will not have a substantial impact on gender equity if equal access to quality, full-time formal employment opportunities, with equal pay for equal work, is not achieved as well [47,48]. Policies and interventions that could help to support women who want to enter or remain in the workforce include job training, minimum wages, paid maternity and/or parental leave, child care, support for female entrepreneurship, and social security. The elimination of gender discrimination and sexual harassment at work can also enhance women’s economic advancement [51]. A continued emphasis on increasing girl’s education, such as affordable basic education, reducing distances to school and elimination of child marriage, is fundamental to promoting women’s empowerment and economic mobility [47]. Investments in girls’ education have high returns in terms of both economic and health benefits, including reduced fertility and lower infant and maternal mortality rates [17,47,52].

Improving women’s overall health through better access to quality healthcare, clean water and safe sanitation, and proper nutrition will have long-lasting social and economic benefits for individuals and governments. Ensuring women have control over fertility through access to family planning and safe abortion services can help women to meet their reproductive goals, reduce maternal and infant mortality rates, and promote female achievements in education and labor force participation [47,52]. We should also work to eliminate gender-based violence which causes negative health outcomes, constrains women’s autonomy, and prevents women from reaching their full potential [47,52]. This includes eliminating child marriage, domestic violence, and sex-selective abortions [47,52]. Concerted efforts and commitments from various stakeholders and organization from community, national, and international levels are needed to draw attention to violation of these human rights [5,46].

### Study contributions, strengths, and limitations of the study

We used publicly available data that enabled a low-cost, transparent study. Various trend data have been available for more than a decade, which allows for the examination of the association between outcomes and events over time. For strengthening causal inference, interrupted time-series analysis (ITSA) is stronger than typical ecological studies because it allows researchers to (1) quantify the change in slopes and levels of outcome after the event and (2) compare the post-event trend with that from the average trend of control countries that have similar trend before the event [7,28,30]. The GGI was the best possible index available for this study because it covered 12 annual data points (2006–2017) and created estimates using important subsets of indicators in almost 150 countries with consistent methods [10–21]. The GGI was: (1) constructed using the same methods to capture four important dimensions of gender equity; (2) generated and shared annually from 2006 to 2017, and (3) sensitive to events and societal changes (based on presented analysis in this study). During the time in which we conducted this study, the UNs Gender Inequality Index (GII), Gender Development Index (GDI), and Inequality-adjusted Human Development Index (IHDI) had only six annual data points (2010–2015) [6], which was not adequate for the analysis [7].

Regarding limitations, first, although the GGI has a lot of potential, several considerations are needed in interpreting the data. The GGI represents aggregated, self-reported national data, which may not represent all populations within a country. For example, in rural areas, it is common for women to work in fields and on farms, but these positions are not likely to be counted in labor force participation data. It is also possible that some changes in GGI may result from changes in data reporting. The GGI and its sub-indices should be interpreted alongside other national indicators. For example, Nicaragua did not perform well in economy, education, and health indicators [1,22], but still ranked high in terms of overall gender equity. The GGI is complex and gender equity is often influenced by shifts in cultural norms which can take many years to have measurable impacts on society [10].

Second, we might not able to capture the true effect of the events with the view from outsiders. Due to the pervasiveness of norms and cultural beliefs around gender, key events might have different impact on gender equity within and among countries. For example, employment discrimination or gender-based violence may raise red flags globally but could be considered normal and thus less harmful within the country itself [35]. On the other hand, if those affected perceive the violations as serious but have no means to report or enforce laws against them, it can further reduce gender equity within a country because it demonstrates systemic failures to protect women’s rights [35].

Third, we did not randomly select studied countries from 53 countries with shifts in GGI between 2006 and 2017, which limits the generalizability of the findings. For the purpose of our investigation, we selected countries with diverse geographical location, political, economic, population, and health status [1,22,23]. Countries were also selected based on the experiences of at least one coauthor to allow us to capture aspects that those who were not familiar with might have missed (e.g. important events or local perceptions).

Finally, although we reviewed multiple sources to ensure that relevant events were captured [10–21,23–27], it is also possible that some relevant events were overlooked or that alternative interpretations of the importance of these events on gender equity exist.

## Conclusion

Using interrupted time-series analysis, we found that key social, economic, and political events can help explain trends in countries’ gender equity. Our study suggests that supportive and modifiable social and political environments play an important role in empowering women, which would advance human rights and promote health and well-being of individuals, households, communities, and countries.

## Supplementary Material

Supplemental Material

## Data Availability

The data are publicly available, and the sources are referenced within the manuscript. The data collected from the gender gap index and its sub-indices were summarized in an MS Excel file that will be made publicly available pending acceptance of the manuscript for publication.

## Paper context

Supportive social and political environments are key to ensuring better health and gender equity for women globally. We utilized an innovative analytical method to examine changes in gender equity in China, Nepal, and Nicaragua. Major social, economic, and political events from 2006 to 2017 in these countries are used to explain changes in their Global Gender Gap Index scores. Based on these findings, we propose several recommendations to continue progress in gender equity worldwide.

## References

[1] World Bank. World development indicators. Washington, DC: World Bank Group; 2017. Available from: https://data.worldbank.org/products/wdi

[2] Huynen MM, Martens P, Hilderink HB. The health impacts of globalization: a conceptual framework. Global Health. 2005;1:14. 16078989 10.1186/1744-8603-1-14 PMC1208931

[3] Kumar S, Kumar N, Vivekadhish S. Millennium development goals (MDGs) to sustainable development goals (SDGs): addressing unfinished agenda and strengthening sustainable development and partnership. Indian J Community Med. 2016;41:1–10. 26917865 10.4103/0970-0218.170955 PMC4746946

[4] World Health Organization. Maternal mortality. Geneva: WHO; 2018 [cited 2018 76]. Available from: http://www.who.int/news-room/fact-sheets/detail/maternal-mortality

[5] United Nations. Sustainable development goals report 2016. New York: United Nations; 2016.

[6] United Nations Development Programme. Human development reports: human development data (1990–2015). UNDP; 2018 [cited 2018 321]. Available from: http://hdr.undp.org/en/data

[7] Bernal JL, Cummins S, Gasparrini A. Interrupted time series regression for the evaluation of public health interventions: a tutorial. Int J Epidemiol. 2017;46:348–355. 27283160 10.1093/ije/dyw098 PMC5407170

[8] Michielutte R, Shelton B, Paskett ED, et al. Use of an interrupted time-series design to evaluate a cancer screening program. Health Educ Res. 2000;15:615–623. 11184220 10.1093/her/15.5.615

[9] Cruz M, Gillen DL, Bender M, et al. Assessing health care interventions via an interrupted time series model: study power and design considerations. Stat Med. 2019;38:1734–1752. 30616298 10.1002/sim.8067 PMC7959401

[10] Hausmann R, Tyson LD, Zahidi S. The global gender gap report 2006. Geneva: World Economic Forum; 2006.

[11] Hausmann R, Tyson LD, Zahidi S. The global gender gap report 2007. Geneva: World Economic Forum; 2007.

[12] Hausmann R, Tyson LD, Zahidi S. The global gender gap report 2008. Geneva: World Economic Forum; 2008.

[13] Hausmann R, Tyson LD, Zahidi S. The global gender gap report 2009. Geneva: World Economic Forum; 2009.

[14] Hausmann R, Tyson LD, Zahidi S. The global gender gap report 2010. Geneva: World Economic Forum; 2010.

[15] Hausmann R, Tyson LD, Zahidi S. The global gender gap report 2011. Geneva: World Economic Forum; 2011.

[16] Hausmann R, Tyson LD, Zahidi S. The global gender gap report 2012. Geneva: World Economic Forum; 2012.

[17] Hausmann R, Tyson LD, Forum WE. The global gender gap report 2013. Geneva: World Economic Forum; 2013.

[18] Hausmann R, Tyson LD, Forum WE. The global gender gap report 2014. Geneva: World Economic Forum; 2014.

[19] Hausmann R, Tyson LD, Forum WE. The global gender gap report 2015. Geneva: World Economic Forum; 2015.

[20] Hausmann R, Tyson LD, Forum WE. The global gender gap report 2016. Geneva: World Economic Forum; 2016.

[21] Hausmann R, Tyson LD, Forum WE. The global gender gap report 2017. Geneva: World Economic Forum; 2017.

[22] UNICEF. The state of the world’s children 2017: children in a digital world. New York: UNICEF; 2017.

[23] Central Intelligence Agency. The world factbook. Washington (DC): CIA; 2018.

[24] BBC. China profile timeline: a chronology of key events. BBC; 2017 [cited 2018 312]. Available from: http://www.bbc.com/news/world-asia-pacific-13017882

[25] BBC. Nepal profile timeline: a chronology of key events. BBC; 2017 [cited 2018 312]. Available from: http://www.bbc.com/news/world-south-asia-12499391

[26] BBC. Nicaragua timeline: a chronology of key events. BBC; 2017 [cited 2018 312]. Available from: http://news.bbc.co.uk/2/hi/americas/1225283.stm

[27] World Bank. Where we work. The World Bank; 2018 [cited 2018 1116]. Available from: https://www.worldbank.org/en/where-we-work

[28] Linden A. Conducting interrupted time-series analysis for single- and multiple-group comparisons. Stata J. 2015;15:480–500.

[29] Baum CF, Schaffer ME. A general approach to testing for autocorrelation. Boston: Boston College; 2013.

[30] Linden A. A matching framework to improve causal inference in interrupted time-series analysis. J Eval Clin Pract. 2018 4;24:408–415. 29266646 10.1111/jep.12874

[31] Liu B, Li L, Yang C. Gender equality in China’s economic transformation. New York: United Nations; 2014. p. 21–22.

[32] International Labour Organization. ILO Asia-Pacific working paper series: women in the labour market in China. Bangkok: ILO; 2015.

[33] Catalyst. Women in the workforce: China. 2017 [cited 2018 76]. Available from: http://www.catalyst.org/knowledge/women-workforce-china

[34] Stauffer B. “Only men need apply” gender discrimination in job advertisements in China. New York: Human Rights Watch; 2018.

[35] China Labor Bulletin. Workplace discrimination. China Labor Bulletin; 2019 [updated 8 2019; cited 2019 1029]. Available from: https://clb.org.hk/content/workplace-discrimination

[36] China Ministries and Groups. Further regulating recruitment and promoting women’s employment. Beijing; 2019.

[37] Hesketh T, Lu L, Xing ZW. The consequences of son preference and sex-selective abortion in China and other Asian countries. Cmaj. 2011;183:1374–1377. 21402684 10.1503/cmaj.101368 PMC3168620

[38] Golley J, Tyers R. China’s gender imbalance and its economic performance. The China story. Canberra: The Australian National University; 2012.

[39] Reynolds E The challenge of keeping nepalese girls in school. 2011. Available from: https://www.theguardian.com/global-development/poverty-matters/2011/sep/16/nepal-challenge-keeping-girls-in-school

[40] Human Rights Watch. World report 2017: Nepal events of 2016. Human Rights Watch; 2018 [cited 2018 52]. Available from: https://www.hrw.org/world-report/2017/country-chapters/nepal

[41] UNDP Nepal. The interim constitution of Nepal, 2063 (2007). New York: UNDP; 2009.

[42] Baruah N, Reyes J. Nepal elections: more women have a seat at the table, but will they have a voice? San Francisco: The Asia Foundation; 2017 [cited 2018 76]. Available from: https://asiafoundation.org/2017/12/13/nepal-elections-women-seat-table-will-voice/

[43] Freedom House. Freedom in the world 2018-Nicaragua. New York: Freedom House; 2018.

[44] World Bank. Trends in maternal mortality 2000 to 2017: estimates by WHO, UNICEF, UNFPA, World Bank Group and the United Nations Population Division (Vol. 2). Washington (DC): World Bank Group; 2019.

[45] Capoy A. Nicaragua, the world’s unlikely champion of gender equality. Quartz; 2015 [cited 2019 1024]. Available from: https://qz.com/556722/nicaragua-the-worlds-unlikely-champion-of-gender-equality/

[46] United Nations Women. Progress on the sustainable development goals: the gender snapshot 2019. New York: United Nations; 2019.

[47] Klugman J, Hanmer L, Twigg S, et al. Voice and agency: empowering women and girls for shared prosperity. Washinton, DC: The World Bank; 2014.

[48] International Labour Office. Women at work: trends 2016. Geneva: ILO; 2016.

[49] Milazzo A, Goldstein M. Governance and women’s economic and political participation: power inequalities, formal constraints and norms. Washinton, DC: World Bank; 2017.

[50] Morton M, Klugman J, Hanmer L, et al. Gender at work: a companion to the world development report on jobs. Washington, DC: World Bank; 2014.

[51] United Nations. Leave no one behind: a call to action for gender equality and women’s economic empowerment. New York: United Nations; 2017.

[52] Razavi S. World development report 2012: gender equality and development—a commentary. Dev Change. 2012;43:423–437.

