# Supplementary material for: Social, economic, and political events affect gender equity in China, Nepal, and Nicaragua: a matched, interrupted time-series study
Source: Glob Health Action. 2020 Jan 15;13(1):1712147. doi: 10.1080/16549716.2020.1712147 (PMC7006713; doi:10.1080/16549716.2020.1712147)
Supplement: Supplemental Material [file ZGHA_A_1712147_SM0060.docx]

**Supplementary material**

**Appendix 1.** Countries with major shifts in Gender Gap Index (GGI) between 2006 and 2017 [10-21]

| Albania  Angola  Austria  Bahrain  Belgium  Benin  Bolivia  Botswana  Burkina Faso  Chile  China  Columbia  Cuba  Denmark | Ecuador  El Salvador  Estonia  Ethiopia  Finland  France  Guatemala  Honduras  Hungary  Italia  Kenia  Lesotho  Luxembourg | Madagascar  Malawi  Mali  Mauritania  Moldova  Mongolia  Nepal  Nicaragua  Nigeria  Paraguay  Peru  Saudi Arabia  Senegal | Slovenia  South Africa  Spain  Sri Lanka  Suriname  Swimland  Syria  Tanzania  Trinidad and Tobago  Uganda  United States  Uruguay  Venezuela |
| --- | --- | --- | --- |

**Appendix 2:** General characteristics of China, Nepal, and Nicaragua [1,22,23]

|  | **China** | **Nepal** | **Nicaragua** |
| --- | --- | --- | --- |
| **Population and health** (Data from UNICEF) [22] |  |  |  |
| Population (thousand; 2016): |  |  |  |
| Total population | 1,403,500 | 28,983 | 6,150 |
| Population under 18 years | 295,112 | 11,190 | 2,174 |
| Population under 5 years | 85,866 | 2,756 | 597 |
| Urbanized population (%) | 57 | 19 | 59 |
| Life expectancy at birth (years) | 76 | 70 | 75 |
| Total fertility rate | 1.6 | 2.1 | 2.2 |
| Annual number of births (thousands) | 17,035 | 573 | 121 |
| Infant mortality rate (under 1) | 9 | 28 | 17 |
| Under-5 mortality rate | 10 | 35 | 20 |
| Malnutrition in Under-5 children (%; 2011-2016) |  |  |  |
| Stunting | 8 | 36 | 23 |
| Overweight | 7 | 1 | 6 |
| Wasting | 2 | 10 | 2 |
| Use of basic drinking water services (%; 2015) |  |  |  |
| Total | 96 | 88 | 82 |
| Urban | 96 | 89 | 97 |
| Rural | 96 | 87 | 61 |
| Use of basic sanitation services in (%; 2015) |  |  |  |
| Total | 75 | 46 | 76 |
| Urban | 86 | 52 | 86 |
| Rural | 61 | 45 | 63 |
| Total adult literacy rate (%; 2011−2016) | 95 | 60 | 78 |
|  |  |  |  |
| **Economy and labor (2017)** (Data from World Bank)[1] |  |  |  |
| Economy classification | Upper-Middle | Low | Lower-middle |
| GDP per capita (current US$) | 8759 | 901 | 2168 |
| GDP per capita, PPP (current international $) | 16782 | 2867 | 5687 |
| Labor force participation rate, female (% of female population ages 15-64) (modeled ILO estimate) | 69 | 84 | 53 |
| Labor force participation rate, male (% of male population ages 15-64) (modeled ILO estimate) | 83 | 86 | 86 |
| Labor force participation rate, total (% of total population ages 15-64) (modeled ILO estimate) | 76 | 85 | 69 |
| Land area (sq. km) | 9388210 | 143350 | 120340 |
|  |  |  |  |
| **Political and Social** (Data from CIA Factbook) [23] |  |  |  |
| Government type (2019) | Communist party-led state | Federal parliamentary republic | Presidential republic |
| Ethnic groups (list the ones with at least 5%) | Han Chinese 91.6% (2010 est.) | Chhettri 16.6%, Brahman-Hill 12.2%, Magar 7.1%, Tharu 6.6%, Tamang 5.8%, Newar 5%, Kami (2011 est.) | Mestizo (mixed Amerindian and white) 69%, white 17%, black 9%, Amerindian 5% |
| Religions (list the ones with at least 5%) |  |  |  |
|  | Buddhist 18.2%, Christian 5.1%, folk religion 21.9%, unaffiliated 52.2% (2010 est.) | Hindu 81.3%, Buddhist 9%, (2011 est.) | Roman Catholic 50%, Evangelical 33.2%, unspecified 13.2%, (2017 est.) |

**Appendix 3.** Major events and associated types (e.g., Educational (Edu), Health (Hea), Economic (Eco), Political (Pol), and Other (Oth)) in the studied countries [10-21,23-27].

| **Year** | **China** |  | **Nepal** |  | **Nicaragua** |  |
| --- | --- | --- | --- | --- | --- | --- |
|  | **Events** | **Type** | **Events** | **Type** | **Events** | **Type** |
| **2006** | Serious drought affected 18 million people.  Government admitted pollution degraded China's environment and threatened health and social stability. | Eco  Hea | King Gyanendra agrees to restore parliament.  The government signs a peace deal with the Maoists to end the decade-long rebellion. | Pol | Free trade deal with the US comes into effect.  Tough new bill that bans abortions, including in cases where the mother's life is at risk.  Ex-president is returned to power. | Eco  Hea  Pol |
| **2007** | New labor law introduced to protect labors from exploitation.  Food and drug scandals caused concerns about the safety of Chinese exports. | Eco  Hea | Maoists join, quit, and rejoin government.  Parliament approves the abolition of monarchy. | Pol | The International Court of Justice in the Hague settles a long-running territorial dispute between Honduras and Nicaragua. | Pol |
| **2008** | Anti-China protests escalated in Tibet.  A massive earthquake hits Sichuan province, killing tens of thousands.  Beijing hosts Olympic Games.  Tainted milk scandal made ~53,000 Chinese children fall ill.  Global financial crisis affected China. The government announces a $586bn stimulus package to avoid the economy slowing. | Pol  Eco  Hea | Maoists win the largest bloc of seats in elections.  Nepal becomes a republic. Coalition government formed. | Pol |  |  |
| **2009** | Ethnic violence in the restive Xinjiang region.  First sign of relaxation of strictly enforced one-child policy to counter effects of ageing population. | Pol  Hea | Prime Minister and President resign.  Integration of former rebel fighters into the military.  Maoist-led land grab in the far west. Rise to fears for peace process. | Pol | Constitutional Court lifts ban on president seeking re-election. | Pol |
| **2010** | 17.7% rise in exports to become the world's biggest exporter.  Google ends its compliance with Chinese internet censorship. | Eco | The Constituent Assembly votes to extend the deadline for drafting the constitution. | Pol |  |  |
| **2011** | China became the world's second-largest economy. | Eco  Pol | United Nations ends its peace monitoring mission. | Pol | The President is re-elected for another five-year term. | Pol |
| **2012** | Rural-to-urban migrations increased.  Imports and exports decreased, global economic slowdown had effects on economic growth.  Communist Party holds congress expected to start a transfer of power to a new generation of leaders. | Eco  Pol | The Constituent Assembly is dissolved. | Pol | 3,000 people evacuated from areas near the erupting San Cristobal volcano. | Eco  Edu Hea |
| **2013** | Xi Jinping became president. He launches an efficiency and anti-corruption drive.  Former senior leader Bo Xilai is sentenced to life in prison for bribery, misuse and abuse of power. | Pol | The left-wing Nepali Congress wins the second Constituent Assembly elections. No party with a majority. | Pol | Congress approves a proposal for a canal linking the Pacific and the Atlantic Oceans which would rival the Panama Canal. | Eco |
| **2014** | China's trade surplus reaches $31.9bn (increased 14% from the previous year).  Protests Beijing's plans to vet candidates for elections in 2017 grip Hong Kong. | Eco  Pol | New elected prime minister.  Nepal and India sign a deal to build a $1bn hydropower plant on Nepal's Arun river to counter crippling energy shortages. | Pol  Eco | Changes to Nicaragua's constitution come into effect.  Nicaraguan officials announce that construction of a new $50bn canal linking the Atlantic and the Pacific oceans. | Pol  Eco |
| **2015** | The Communist Party announces the end the one-child policy. | Hea | A 7.8-magnitude earthquake strikes Kathmandu and its surrounding areas.  Parliament passes a landmark constitution. | Eco  Edu  Hea Pol | The International Court of Justice rules in favor of Costa Rica in its long-standing border dispute with Nicaragua. | Pol |
| **2016** | Economic growth in 2015 falls to lowest rate in 25 years (6.9%, down from 7.3% in 2014). | Eco | Reduce tension between the Government and ethnic minority Madhesi communities.  Maoist party pulls out of the governing coalition. Prime Minister resigns. Maoist party leader elected as prime minister. | Pol | The government frees 8,000 prisoners to ease overcrowding in Nicaragua's jails.  The president is reelected. | Pol |
| **2017** | Congress remove a two-term limit on the presidency from the constitution. | Pol | Three former soldiers are convicted over the murder of a 15-year-old girl during the civil war.  China and Nepal hold a joint military exercise. | Pol |  |  |
